# Goodbye China: What Do Fewer Foreigners Mean for Multinationals and the Chinese Economy?

**DOI:** 10.1007/s10272-022-1075-0

**Published:** 2022-10-10

**Authors:** Frank Bickenbach, Wan-Hsin Liu

**Affiliations:** Kiel Centre for Globalization (KCG), Kiel Institute for the World Economy (IfW), Kiellinie 66, 24105 Kiel, Germany

**Keywords:** J61, F23

## Abstract

The number of foreigners living in China is very low in international comparison and has further declined recently. While the strict COVID-19-related travel restrictions played a major role in this decline, there are indications that the decline started in part before the pandemic and may well continue once the pandemic-related restrictions are lifted. Against this background, this article discusses the economic challenges that the reduction in the number of foreigners is causing for Western multinationals operating in China and to the Chinese economy more generally. The consequences could spill over to the world economy and reinforce economic and technological decoupling tendencies between China and the West.

According to preliminary results of the 2020 National Census of China, there were 845,697 foreigners and an additional 584,998 residents of Hong Kong, Macao and Taiwan – together 1,430,695 immigrants[1] – living in mainland China on 1 November 2020 (NBSC, 2021). This was a substantial increase of 410,550 immigrants (40.2%) compared to the previous Census of 2010. The increase was greater for foreigners (251,865; 42.4%) than for residents of Hong Kong, Macao and Taiwan (158,685; 37.2%). Still, the total number of immigrants amounted to just 0.1% (and that of foreigners to just 0.06%) of China's population of more than 1.41 billion people.[2] This is an exceptionally low share not only compared to Western countries such as the United States (15.4%), Germany (15.7%) or France (12.8%) but also compared to China's East Asian neighbours Japan (2.0%) or South Korea (2.3%), or even compared to the similarly populous – but economically less developed – India (0.4%) (UNDESA, 2022).

In addition, the overall immigration growth rate of more than 40% from 2010 to 2020 is the result of very heterogeneous developments across Chinese provinces. While the number of immigrants in Yunnan Province in South China has increased by about 700% (from 47,396 to 379,281) between 2010 and 2020,[3] the number of immigrants has actually decreased in several other provinces. This is true, in particular, for the municipalities Shanghai and Beijing where the number of immigrants has decreased by about 21.4% (from 208,602 to 163,954) and 41.5% (from 107,445 to 62,812), respectively. And, although the preliminary results of the 2020 Census published so far do not provide the numbers of foreigners for individual provinces, given the exceptional development of the number of immigrants in Yunnan, it can reasonably be assumed that the aggregate number of foreigners in the other 30 Chinese provinces has actually declined between 2010 and 2020 by roughly 80,000 persons or about 14.5%, according to a back of the envelope estimation.[4]

1 Immigrants in this paper include residents from Hong Kong, Macao and Taiwan as well as foreigners from outside the Greater China region living in mainland China.

2 This figure refers to mainland Chinese living in China, i.e. excluding immigrants in mainland China.

3 In Yunnan, the large majority of foreigners are migrant workers from neighbouring countries Vietnam, Myanmar and Laos, who have been attracted by large disparities in labour markets between Yunnan and these neighbouring countries. In addition, there has been a booming cross-border marriage market, attracting many migrant women to Yunnan. Percentage of immigrants in Yunnan as a share of all immigrants in China has increased from less than 5% in 2010 to about 27% in 2020.

4 The estimation assumes that the number of residents from Hong Kong, Macao and Taiwan in Yunnan has grown at the same rate as for China as a whole (37.2%).

As the National Census was carried out in late 2020, it has already been affected by the COVID-19 pandemic and the strict travel restrictions imposed in response (see below). By this time, an unknown number of immigrants had already left China and/or were unable to enter or return to China due to these strict travel restrictions. Given that foreigners, including expatriates from Western countries, have more likely been directly affected by these restrictions than residents from Hong Kong, Macao and Taiwan, these restrictions have certainly contributed to the particularly strong decrease in the number of immigrants in Beijing and Shanghai, where the number of foreigners has been substantially higher than the number of residents from Hong Kong, Macao and Taiwan.[5]

Additionally, there is evidence showing that the decade-long increase in the number of foreigners living in China had already come to a halt or even reversed in China (outside Yunnan) several years before the COVID-19 pandemic. Take Shanghai, the most international city in China, as an example. While the number of foreigners increased substantially from 100,011 in 2005 to 174,192 persons in 2012 (roughly 8% annual growth rate); it only increased to 178,335 persons in 2015 (roughly 0.8% annual growth rate) and then decreased to 172,076 in 2018 (SBS, 2014-2019). A similar trend reversal can be observed among German citizens living in China. According to the 2010 Population Census, Germans were the second largest group of Europeans living in China in 2010 (NBSC, 2012). According to the German Statistical Office, in each year from 2001 to 2014, the number of German citizens moving from Germany to China exceeded the number of Germans re-migrating from China to Germany, resulting in a net migration of 6,325 Germans to China between 2001 and 2014 (Destatis, 2022). In contrast, in each year between 2015 and 2020, more Germans moved back to Germany from China than the other way around, leading to a net remigration of 4,159 Germans back from China to Germany during this period.

## China's immigration policy

Given its large population and its developing economic status, China does not perceive itself as a potential immigration country. Accordingly, the development of China's immigration policy has been the result of a perceived need for China to control and regulate increasing immigration flows rather than attempt to actively promote immigration or facilitate the integration of immigrants into the Chinese economy and society.

China's Exit and Entry Administration Law, which was passed in 2012, marked an important turning point in China's immigration policy. Over the more than three decades from the beginning of China's open-door policy to the 2012 law, China's legal and administration framework for immigration control and management had been highly fragmented and lacked coordination (Zhu and Price, 2013; Pieke, 2014). A large number of central and local authorities were involved in entry procedures, and the communication and cooperation between them was highly deficient. This framework was inadequate to cope with the economic, social and security challenges resulting from the surging number of legal and illegal immigrants that followed China's access to the World Trade Organization in 2001 (Zhu and Price, 2013). The 2012 law reflected the Chinese government's recognition that China needed a more comprehensive regulatory framework for immigration in order to further its national development goals. It provides for an immigration policy that strongly differentiates the treatment of foreigners by their perceived benefits to the Chinese economy. While particularly highly skilled foreigners are wanted and welcome given their potential benefits for China's economic development, the entry of illegal or less desirable foreigners and their stay in China are to be more strictly controlled and countered than before in the interest of national and social security.

To be sure, China's strong preference for attracting high-skilled immigrants was not first established by the 2012 law. Already in 2004, the Measures for the Administration of Examination and Approval of Foreigners' Permanent Residence in China gave preference to foreigners whose qualifications meet certain pre-determined criteria. In 2008, the Thousand Talents Program was initiated to actively attract highly skilled professionals for advanced research, innovation and the establishment of high-tech start-ups in China. Since at that time the criteria for the so-called preferred high-skill professionals were still very high and the policy measures were carried out only selectively without comprehensive coordination and supplementary hands-on measures, their impact on attracting foreigners, especially those without an ethnic Chinese background, were rather limited (Wang, 2012). Since the 2012 law, the scope of the related policies to attract high-skilled foreigners has become broader, reflecting President Xi Jinping's repeated emphasis of the importance of these talents to support China's innovation-driven economic development. The rules and regulations related to immigration were further consolidated and focused in favour of highly qualified foreigners with the introduction of the Permit for Foreigners to Work in China in 2015. This regulation, which was implemented nationwide in 2017, formally established a strict grading system for foreigners

5 In Beijing and Shanghai, the shares of foreigners were about 84.8% and 68.8%, respectively, of the total number of immigrants in 2010 (NBSC, 2012).

to help China more effectively attract international professionals in line with domestic demand.

Also in 2015, the Chinese authorities started to implement measures to ease the visa and residence application procedures and expanded immigration services for targeted foreign professionals/high-skilled foreign nationals, at first on an experimental basis in a number of selected pilot free trade and demonstration zones. In August 2019, several of these policies (relating to the facilitation of visas and residence permits, including long-term or permanent residence permits for specific high-level foreign talent and the establishment of immigration service centres) have been implemented nationwide to encourage, support and facilitate foreign talent, outstanding young foreigners and overseas Chinese to start businesses, invest, innovate, study and work in China (Ministry of Public Security, 2019).

In view of the consolidation of the legal framework and the (selected) facilitation of visas and residence approvals, China's immigration policy has become overall more favourable for particularly high-skilled foreigners since 2012. By contrast, for most less skilled migrants, Chinese immigration policies have become rather more restrictive due to stricter enforcement of the restrictive entry and residence regulation for this group. At the same time, Chinese public attitudes towards foreigners have become more divided, and public resentment towards foreigners has been on the rise since about 2015 (Speelman, 2020). Against this background, in 2020 the announcement of a public consultation on the Regulations Governing the Permanent Residence of Foreigners, which is intended to better regulate and also ease foreigner's permanent stay in China, met with strong headwinds and harsh criticism from the public. These strong public reactions, particularly on social media, even forced the responsible authorities to offer public assurance that the regulations will be further "improved" and will not be enforced hastily.

### COVID-19-based travel restrictions and the decline in the number of foreigners

Since March 2020, China has implemented strict travel restrictions to deal with the threat of the COVID-19 pandemic. The restrictions imposed included a drastic reduction in the number of international passenger flights in and out of China, broad restrictions on the issuance of visas combined with more complex and time-consuming application procedures than before, strict vaccination requirements and stringent COVID-19 testing and quarantine requirements both prior to and after arrival in China. Despite some adaptations in response to the changing global pandemic situation, strict travel restrictions have remained in place at least until mid-2022 (the time of writing).

As a consequence, the number of people, and in particular the number of foreigners, crossing China's border decreased dramatically over the past two years. The number of border crossings by foreigners decreased from 97.7 million in 2019 to 13.2 million in 2020 and to a mere 4.5 million (-95.4%) in 2021 (NIA, 2020, 2022). In light of these developments, the number of foreigners living in China has likely further declined since the reference date of the 2020 National Census (1 November 2020). And given the continuation of COVID-19-related restrictions and the COVID-19 outbreaks and related strict lockdowns in, for example, Jilin and Shanghai in spring 2022, it is likely that the number of foreigners living in China will decline even further in the near future. In a recent survey among foreigners in Shanghai conducted during the strict and long lockdown of the municipality in April 2022, 85% of the 950 respondents said that the lockdown has made them rethink their future in China. Furthermore, 22% and 26%)of respondents said they want to leave China as soon as possible or within 12 months, respectively. Only 15% of respondents still planned to stay in China for the long haul (That's Shanghai Magazine, 2022).

### Implications for Western multinationals in China

The COVID-19-related travel restrictions and the associated decline in the number of foreigners in China have had significant negative consequences for many Western companies. Business surveys conducted by various chambers of commerce in China in 2021 have found that travel and entry restrictions due to the COVID-19 pandemic and related human resource challenges were by then considered to be among the most pressing operational business challenges for Western companies active in China. British companies in China surveyed in autumn 2021, for example, rated "employing foreign staff" the most burdensome challenge for doing business in China in 2021. At the time of the survey, 43% of the British companies had existing or new foreign employees outside China that were unable to enter the Chinese mainland due to COVID-19-related problems of booking suitable flights, securing visas for themselves or their dependents or because they were unwilling to quarantine (British Chamber, 2022).

The travel and entry restrictions have significantly exacerbated the problems many Western companies in China face in attracting and retaining foreign talent. Among the European companies surveyed in February 2021, more than 60% of respondents ranked "entry restrictions into China due to COVID-19" as one of their top three challenges for attracting and for retaining international talent in

China at that time (European Chamber, 2021a). More than three quarters (and almost one-third) of American companies in China surveyed in autumn 2021 considered it a significant challenge for retaining and recruiting international talent in China that qualified candidates were unable (and unwilling, respectively) to move to China due to the COVID-19 restrictions (AmCham China, 2021).

The COVID-19-related travel and entry restrictions have thus led to a reduction of the number (shares) of foreign staff employed by Western companies in China. For example, in autumn 2021, 23% of British companies surveyed reported a net decrease (vs. only 3% reported a net increase) in foreign employees over the past year (British Chamber, 2021). For 2022, 41% of British companies expected that "a significant number of foreign employees will leave China indefinitely", mainly due to emotional strain of prolonged separation from friends and family or challenging travel requirements and logistics (British Chamber, 2021, 23). US companies in China reported an even stronger impact of COVID-19 restrictions on foreign employment. In late March 2022, about 28% of US firms surveyed stated that they had reduced their foreign staff in China due to COVID-19 restrictions by more than 20% since the beginning of the pandemic, and another 13% of firms by between 10% and 20%. About 44% of companies expected a further loss of foreign staff, if "current COVID-19 restrictions remain in place for the next year" (AmCham China and AmCham Shanghai, 2022).

In line with the general decline in the number of foreigners in China, the reduction in the number or share of foreign employees in many Western companies in China did not just start with the pandemic. According to the German Chamber (2021), for example, the average percentage of foreign employees in German companies in China had been declining well before the outbreak of the pandemic, decreasing from 7.1% in 2016 to 6.3% in 2019 and further to 5.4% in 2021. When asked about the reasons for replacing foreign employees with local employees, 30% of responding firms named COVID-19 visa restrictions and 26% the visa process in general. However, also about 60% of responding firms named the wage level, about 37% better business contacts and 35% (high) qualifications of local employees (German Chamber, 2021).[6]

Given the difficulties that companies currently encounter in attracting and retaining foreign employees, the decline in the number of foreign employees will likely continue. And as the reasons for these difficulties are not limited to pandemic-related restrictions, the decline is likely to continue even after a (future) lifting of these restrictions. According to the European and American companies surveyed, those reasons include the unwillingness of qualified candidates to relocate to China, high expectations of salary or high costs of living and payroll costs, a lack of affordable, quality education for children, poor air quality, internet/media restrictions, and importantly in the case of American companies also bilateral tensions and geopolitical concerns (European Chamber, 2021a; AmCham China, 2022).

Despite COVID-19-related travel restrictions and increasing difficulties in retaining and recruiting foreign professionals to support their business operations in China, Western companies surveyed generally continue to view China as a key market and an attractive investment location for the near future. In autumn 2021, almost half of British companies surveyed and a large majority of US and German companies planned to increase their investment in China over the next year or two (British Chamber, 2021; AmCham China, 2022; German Chamber, 2022).[7] Rather than reducing their China engagement, many companies have apparently responded to the challenges by further increasing their localisation efforts. For some time now, the majority of European companies operating in China have been producing in China primarily for the Chinese market and have increasingly been relocating their procurement (supply chains) and in some cases also their R&D for the Chinese market to China (European Chamber, 2021b). In recent years, many companies have further intensified their localisation efforts to cope with economic and technological decoupling tendencies spurred by political tensions between China and mainly the US but also Europe (European Chamber, 2021b; German Chamber, 2022). COVID-19-related travel restrictions appear to have reinforced this localisation trend by (further) increasing local sourcing and local R&D activities, and transferring technical and operational know-how and decision-making power to the Chinese subsidiaries (German Chamber, 2022b).

6 About one quarter of respondents named the Individual Income Tax (IIT) reform, which was planned to take effect on 1 January 2022, but was postponed by two years in December 2021. The reform stipulated the elimination of tax exemptions for certain benefits for foreigners (e.g. for housing expenses, children's education expenses, language training expenses and home leave expenses), which would have resulted in larger tax liabilities for many foreign employees.

7 The Omicron outbreaks and subsequent strict lockdowns in Shanghai and several other Chinese provinces in spring 2022 seem to have soured the investment plans of US companies in China, however. By end of March 2022, 17% (29%) of the respondents to a small-scale survey of American companies in China stated that the recent Omicron outbreak led to a decrease (delay) of planned investments. About 50% of companies said they would reduce investments if the current COVID-19 restrictions remain in place for the next year (AmCham China and AmCham Shanghai, 2022).

Overall, the survey results suggest that the replacement of foreign by local employees is in part a natural, efficiency-enhancing process, in which Western firms take advantage of the growing reservoir of highly qualified Chinese workers with well-established local connections to reduce (labour) costs and leverage the specific knowledge and connections of local workers. The results also suggest, however, that many companies are being forced to accelerate this process – beyond what they consider optimal – due to significant difficulties in attracting and retaining foreign employees. In this case, the (forced) replacement of foreign experts by local talent may lead to substantial efficiency losses, e.g. in terms of international coordination and knowledge transfer within the multinational company. Although the number of foreign employees in Western companies in China is generally relatively small, they usually hold critical positions that require special skills, and they are of great importance in terms of corporate culture and the diversity of views in decision-making processes (European Chamber, 2021a).

### Implications for the Chinese economy

China's long-term development goal as emphasised in the 14th Five-Year Plan for 2021-2025 is to promote high-quality development that should be innovation-driven and predominantly fuelled by the Chinese domestic market. Two cornerstones of this new development strategy thus are: first, promoting China's self-reliance and self-improvement in science and technology, and second, strengthening China's domestic economy and promoting self-contained domestic supply chains (Bickenbach and Liu, 2021).

The observed increase of local sourcing and local R&D activities of Western companies over the past years and the amplification of these localisation trends by the COVID-19-related travel restrictions may, at least on the surface, be in line with the goals emphasised in the Five-Year Plan. Other consequences, however, run rather counter to the stated goals of the Chinese leadership. The loss of foreign experts and China's reduced attractiveness to foreign talent as well as a decrease in knowledge exchange with headquarters abroad that was observed by many Western companies (e.g. German Chamber, 2022) are expected to have a negative impact on China's innovation capacity and development. This will be felt not only by foreign companies operating in China but also by domestic Chinese companies and the Chinese economy as a whole, including through reduced knowledge spillovers and learning effects.

In several key areas, Chinese companies are currently still far from the technological leadership that the Chinese government seeks. The desired greater self-reliance in science and technology therefore does not mean that China does not want (or need) to continue to benefit from foreign knowledge. In addition to an increased promotion of its own research and innovation, China will continue to seek knowledge and technology transfers from abroad. According to the Five-Year Plan, China plans to increase its efforts to attract technology-leading foreign companies and their research activities, as well as foreign scientific and technological talent. In addition, Chinese direct investment abroad and targeted acquisitions of leading foreign companies in knowledge- and technology-intensive industries are also likely to continue to be pursued (Xia and Liu, 2021).

Similarly, China's aim of strengthening the domestic market and promoting self-contained domestic supply chains should not be interpreted as a general retreat from promoting foreign trade and investment. The Chinese government also aims to further strengthen China's position as a leading trading power and to foster its development towards a manufacturing superpower. For many specific technologically and qualitatively demanding (intermediate) products, companies in China are currently still dependent on imports and suppliers from outside China. China will therefore further need foreign trade and investments and the transfer of foreign knowledge to achieve its goals.

Worldwide, the COVID-19 pandemic and governments' policies to contain the disease have led to a sharp decline in international trade and foreign direct investment (FDI) in the first half of 2020. Due, in particular, to a relatively successful containment of COVID-19 infections in China, the Chinese economy recovered earlier than many other economies. China thereby contributed substantially to the recovery of global trade and foreign investment flows, which surpassed their pre-pandemic levels already in 2021 (UNCTAD, 2022a, 2022b). Now, there is a serious risk, however, that the further development of the Chinese economy and particularly its foreign trade and investments will be impeded by China's continuing travel restrictions and recurring lockdowns, and in a more long-term perspective by a continuing decline in the number of foreigners in China.

While the negative short-term economic effects of the strict travel restrictions and lockdowns are already apparent (NBSC, 2022), it is still too early to empirically assess the longer-term economic impact of fewer foreigners living in China. The general literature on the relation between immigration and trade, investment and innovation clearly suggests, however, that the reduced number of foreigners in China is likely to have a negative impact on

China's foreign trade and investment as well as on its innovation and growth.

A large number of empirical studies have clearly confirmed that international migration and the presence of foreigners increase international trade and FDI (Hatzigeorgiou and Lodefalk, 2021).[8] Immigrants boost demand for products or services from their home country, which is satisfied by increased imports or by companies from the migrants' home country that invest and produce these products in the migrants' host country. In addition, and more importantly in general, migrants can facilitate and promote international trade and investment by reducing information and transaction costs. They can provide information about products, preferences, business practices, etc. in foreign markets, improve communication between host and home countries, or reduce the costs and uncertainties of negotiating and enforcing contracts between trade or investment partners in the two countries (Hatzigeorgiou and Lodefalk, 2021; Cuadros et al., 2019). Overall, the relevant empirical literature confirms significant positive effects of immigration on both bilateral trade (imports as well as exports) and bilateral FDI (inward as well as outward FDI) between migrants' host and home countries (Hatzigeorgiou and Lodefalk, 2021). As for the relevance of personal characteristics of migrants, it seems that it is mainly migrants' occupations rather than their education levels that determine their effect on trade and FDI. In particular, migrants in occupations that are more closely related to business decisions, such as managers and, to a lesser extent, professionals seem to have a positive impact on trade (Martin-Montaner et al., 2014) and FDI (Cuadros et al., 2019).

International migration is generally also advantageous for fostering innovation in the host countries (Breschi et al., 2016; Venturini et al., 2018). High-skilled immigrants bring with them skills and knowledge that are generally in high demand in the host countries and provide complementary inputs that are crucial for knowledge creation and innovation (Stephan and Levin, 2001). Their different cultural backgrounds increase local cultural diversity which fosters creativity and has a positive impact on patenting and innovation and entrepreneurship, especially in knowledge- or technology-intensive sectors (e.g. Rodríguez-Pose and Hardy, 2015; Niebuhr, 2010). The generally more risk-loving attitude of migrants (Huber and Nowotny, 2020) is an additional feature favouring the uptake of innovation and entrepreneurial activities. The presence of foreign managers and experts in the local subsidiaries of foreign multinationals facilitates an international knowledge transfer within the multinationals and increasing productivity in the subsidiaries (Golob Šušteršič and Zajc Kejžar, 2019), which may then spill over to local firms particularly with increasing localisation of R&D activities of the multinationals.[9]

In light of these general findings, fewer foreigners living in China may mean that China misses opportunities to further intensify its international engagement via trade and investments, to speed up innovation and to achieve its ambitious growth and development goals.

## Conclusions

In several Chinese provinces, including some of its most international ones like Beijing and Shanghai, the number of immigrants has declined in 2020 compared to 2010. Over the more than two years of pandemic-related travel restrictions, the number of foreigners crossing China's border has decreased dramatically and the number of foreigners permanently living in China has further declined. Given the continuation of strict COVID-19 restrictions, China's selective and still quite restrictive migration policy, and the increasing resentment among the public toward foreigners, the number of foreigners living in China may well decline even further in the future.

The travel restrictions and declining foreign workforce have posed huge challenges for both Western multinationals operating in China and the Chinese economy as a whole. Many multinationals appear to have responded to these challenges by further increasing their localisation efforts, partially rather out of necessity and at the expense of firm performance. For China as a whole, the declining number of foreign experts means that the country may miss opportunities for beneficial foreign trade and investments as well as technology transfer and innovation, leading to losses in growth and development potential.

The effects of China's declining foreign workforce and foreign population more generally are not confined to the Chinese economy exclusively, however. They are ex-

8 Most of the studies on the relation between immigration and trade and immigration and FDI relate to the effects of immigration to North American or European countries. The validity of the results for immigration to China is therefore empirically still an open question. From a theoretical perspective, however, the trade and investment promoting effects of migrants are expected to be larger for countries that are less similar culturally and for less developed countries, where institutions are weaker and doing business with foreigners entails a higher degree of insecurity. Migrants may therefore be expected to have a particularly large impact on Western companies' trade and investment relations with China.

9 Most of the studies on the migration-innovation nexus focus on the US and Europe as host countries. Findings from Bahar et al. (2022) suggest, however, that the positive innovation impact of international migration is not limited to the developed countries, but have also helped to increase innovation in emerging economies.

pected to spill over to the world economy, above all, via its negative impact on international trade and investment. Europe, with its many companies operating in China and intensive trade and investment relations, is likely to be affected particularly strongly. The "people-to-people decoupling" spurred by travel restrictions and the declining number of foreigners in China more generally could become another amplifier of the more general economic and technological decoupling tendencies between China and the West. And it will further reduce mutual understanding between China and the West not only in business but also in politics and society at large.
